# Sleep disturbances in Wolfram syndrome

**DOI:** 10.1186/s13023-019-1160-z

**Published:** 2019-08-02

**Authors:** Amy Licis, Gabriel Davis, Sarah A. Eisenstein, Heather M. Lugar, Tamara Hershey

**Affiliations:** 1 Department of Neurology, Washington University School of Medicine, Campus Box 8111, 660 South Euclid Ave, St Louis, MO 63110 USA; 2 Department of Psychiatry, Washington University School of Medicine, Campus Box 8134, 4525 Scott Avenue, St Louis, MO 63110 USA; 3 Department of Radiology, Washington University School of Medicine, Campus Box 8134, 4525 Scott Avenue, St Louis, MO 63110 USA; 4 Department of Psychiatry, Washington University in St. Louis, Campus Box 8134, 4525 Scott Avenue, St Louis, MO 63110 USA; 5 College of Medicine, SUNY Downstate College of Medicine, 450 Clarkson Ave, Brooklyn, NY 11203 USA

**Keywords:** Wolfram syndrome, Sleep disorders, Actigraphy, Sleep studies

## Abstract

**Background:**

Wolfram syndrome is a rare disorder associated with diabetes mellitus, diabetes insipidus, optic nerve atrophy, hearing and vision loss, and neurodegeneration. Sleep complaints are common but have not been studied with objective measures. Our goal was to assess rates of sleep apnea and objective and self-reported measures of sleep quality, and to determine the relationship of sleep pathology to other clinical variables in Wolfram syndrome patients.

**Methods:**

Genetically confirmed Wolfram syndrome patients were evaluated at the 2015 and 2016 Washington University Wolfram Syndrome Research Clinics. Patients wore an actigraphy device and a type III ambulatory sleep study device and completed the Epworth Sleepiness Scale (ESS), the Pittsburgh Sleep Quality Index (PSQI) and/or the Pediatric Sleep Questionnaire (PSQ). PSQI and PSQ questionnaire data were compared to a previously collected group of controls. Patients were characterized clinically with the Wolfram Unified Rating Scale (WURS) and a subset underwent magnetic resonance imaging (MRI) for brain volume measurements.

**Results:**

Twenty-one patients were evaluated ranging from age 8.9–29.7 years. Five of 17 (29%) adult patients fit the criteria for obstructive sleep apnea (OSA; apnea-hypopnea index [AHI] ≥ 5) and all 4 of 4 (100%) children aged 12 years or younger fit the criteria for obstructive sleep apnea (AHI’s ≥ 1). Higher AHI was related to greater disease severity (higher WURS Physical scores). Higher mixed apnea scores were related to lower brainstem and cerebellar volumes. Patients’ scores on the PSQ were higher than those of controls, indicating greater severity of childhood obstructive sleep-related breathing disorders.

**Conclusions:**

Wolfram syndrome patients had a high rate of OSA. Further study would be needed to assess how these symptoms change over time. Addressing sleep disorders in Wolfram syndrome patients would likely improve their overall health and quality of life.

## Background

Wolfram syndrome is a rare autosomal recessive disorder that is caused by mutations in the *WFS1* or, less commonly, the *WFS2* gene [1, 2]. *WFS1* encodes an endoplasmic reticulum protein wolframin [1], which is thought to play a role in protection against ER stress-related apoptosis [3]. The clinical manifestations of Wolfram syndrome can include childhood onset of diabetes mellitus, diabetes insipidus, optic nerve atrophy, hearing and vision loss, motor impairment, and neurodegeneration [4].

Clinically defined classic Wolfram syndrome has been associated with a limited lifespan, and causes of death may have included central or obstructive sleep apnea. In a case series of 45 patients with the classic manifestations of diabetes insipidus, diabetes mellitus, optic atrophy, and deafness (DIDMOAD), the median age of death was 30 years (range 25–49 years) and central respiratory failure with brainstem atrophy was noted as a cause [5]. In another series of 68 patients also defined by the clinical manifestations of DIDMOAD, more than 50% of the 23 patients who died had symptoms of significant neurodegeneration, including apneic spells [6]. Now that genetic identification of Wolfram syndrome is possible, it is apparent that the clinical phenotype is broader than previously described [7]. Thus, the natural history of respiratory issues and their severity across the continuum of the disease phenotype is unclear.

Understanding the nature of sleep dysfunction in Wolfram syndrome has implications for the health and potentially the longevity of patients. Sleep complaints are common in Wolfram syndrome patients and are associated with their overall quality of life, but have not been well-characterized by sleep studies or actigraphy [8]. The presence or severity of sleep dysfunction could be related to the known regional neuropathology of Wolfram syndrome. Wolfram syndrome is associated with decreased brainstem (especially the ventral pons) and cerebellar volumes, among other regions, compared to controls [9, 10]. These regions also have been shown to be involved in or affected by sleep apnea in humans [11, 12].

The present study was designed to objectively measure sleep quality and assess the presence of obstructive and central sleep apnea in genetically confirmed Wolfram syndrome patients. In addition, we explored whether disease severity and neuropathology as measured by regional brain volumes relate to sleep dysfunction. Studying sleep may provide more information on the natural history of Wolfram syndrome, help us understand any sleep-related health risks including those possibly contributing to mortality, and perhaps identify important targets for intervention.

## Methods

### Patients and study design

Wolfram syndrome patients were recruited through the Washington University Wolfram Syndrome Research Clinic, an annual event to collect data relevant to the natural history of the disease, ongoing since its inception in 2010. Patients were recruited through physician referral and the Washington University Wolfram Syndrome International Registry. For enrollment in the research clinic, patients had to be age 30 years or younger at entry, have genetically confirmed Wolfram syndrome (mutations of the *WFS1* gene) and be willing and able to travel to St. Louis. Individuals with Wolfram syndrome completed a comprehensive series of evaluations and questionnaires across multiple domains (sleep, vision, hearing, urology, cognition, psychiatry, neurology, balance and gait, taste and smell, endocrinology and magnetic resonance imaging [MRI]) during the research clinic. Analyses from subsets of these data have been reported elsewhere [7–10, 13–21].

This paper focuses on sleep data from Wolfram patients in relation to other disease severity and brain volume variables and compares self-reported sleep problems to a previously reported sleep questionnaire dataset from controls. Wolfram patient data reported here were collected from all willing Wolfram clinic participants from 2015 and 2016. These were the clinic years that ambulatory sleep study devices and personnel were made available from the Washington University Sleep Medicine Center. No additional exclusion criteria were applied for sleep assessment participants. Controls were composed of individuals with type 1 diabetes (T1DM) and non-diabetic healthy controls who were recruited through the Pediatric Diabetes Clinic at St. Louis Children’s Hospital and through word-of-mouth [8, 9].

### Sleep assessments

#### Ambulatory sleep study

Wolfram syndrome patients slept in a hotel affiliated with Washington University during their clinic participation and agreed to wear an ambulatory sleep device on one of the nights. Patients in the 2015 clinic wore an ApneaLink™ type III ambulatory sleep study device (ResMed Corporation, Poway, Calif). Patients in the 2016 clinic wore a Phillips Respironics Alice NightOne type III ambulatory sleep study device (Koninklijke Philips N.V.). The ambulatory sleep study devices were placed on patients during evening hours and removed during morning hours by registered sleep technologists (RST) employed by the Washington University Sleep Medicine Center. Data collection was considered adequate if four or more interpretable hours of data were obtained [22]. The ambulatory sleep studies were repeated once within the same clinic year if the initial night of recording showed insufficient data collection or if there were technical issues.

The ambulatory sleep studies were scored initially by a RST and all data was also scored and interpreted by a board-certified sleep physician (AL; American Board of Psychiatry and Neurology with Added Qualification in Sleep Medicine). Sleep studies were scored according to standards established by the American Academy of Sleep Medicine (AASM) Manual for the Scoring of Sleep and Associated Events: Rules, Terminology and Technical Specification version 2.3, including the scoring of obstructive apneas, obstructive hypopneas, and central apneas [23]. The apnea hypopnea index (AHI), obstructive apnea index (OAI), central apnea index (CAI), hypopnea index (HI), mixed apneas index (MAI), and oxygen desaturation index (ODI) were calculated for each ambulatory sleep study based on recording time. Adult scoring criteria was used if the patient was 13 years of age or older. Pediatric scoring criteria was used if the patient was 12 years of age or younger per practice guidelines outlined in the International Classification of Sleep Disorders, 3rd edition [24].

Criteria for the diagnosis of obstructive sleep apnea (OSA) and central sleep apnea (CSA) followed the International Classification of Sleep Disorders (ICSD), 3rd edition. OSA was defined per ICSD criteria as “five or more predominantly obstructive respiratory events (obstructive and mixed apneas, hypopneas, or respiratory effort related arousals [RERAs]) per hour of sleep” noted during a sleep study for adult patients, and per ICSD criteria as “one or more obstructive apneas, mixed apneas, or hypopneas, per hour of sleep” for pediatric patients [24]. Adults with an overall AHI of greater than or equal to 5 events per hour of sleep were classified as having OSA if there was a predominance of obstructive respiratory events, and children with an obstructive AHI of greater than or equal to 1 event per hour of sleep were classified as having OSA [24]. CSA was defined per ICSD criteria as CAI of greater than or equal to 5 events per hour [24].

The AHI has been considered the primary metric for diagnosis of OSA based on clinical and research precedence [25, 26]. The AHI has been determined to be a reliable indicator of OSA severity and also of morbidity and mortality related to OSA, including risk of all-cause mortality, cardiovascular disease, arrhythmias, and incident stroke [25, 26]. Therefore, the AHI was the primary sleep variable chosen for further analyses as described below.

#### Actigraphy

Wolfram syndrome patients wore a Phillips Respironics Actiwatch 2, a type of activity monitor that is similar to a wristwatch and is sensitive to motor activity (Bend, OR). Patients wore the Actiwatches for one night. Actigraphy data was analyzed, including the tracings and the numerical data, noting in particular the following parameters: mean sleep efficiency, mean sleep latency, and mean sleep duration. These parameters were chosen to characterize sleep quality and quantity.

#### Sleep questionnaires

The Epworth Sleepiness Scale (ESS) [27] and Pittsburgh Sleep Quality Index (PSQI) [28] and the Pediatric Sleep Questionnaire (PSQ) [29] were administered via a web-based data collection tool (REDCap) within a few months prior to the patients’ arrival at the clinic [30]. The ESS is designed to assess degree of sleepiness, with questions asking the likelihood of falling asleep in eight different conditions rated on a four-point Likert-type scale (0 = never, 3 = high chance) (range of scores 0–24, > 10 is considered abnormal) [27]. The PSQI is designed to assess sleep disturbances, with questions asking the extent to which various factors interfered with sleep on a four-point Likert-type scale (0 = not at all, 3 = three or more times a week), with subscales of subjective sleep quality, sleep latency, sleep duration, habitual sleep efficiency, sleep disturbances, use of sleeping medication, and daytime dysfunction (range of scores 0–21, > 5 is considered abnormal) [28]. The PSQ assesses symptoms of childhood obstructive sleep-related breathing disorders (SRBDs) and includes snoring, sleepiness, and behavior subscales (range of scores 0–1, > 0.33 is considered abnormal) [29]. For the Wolfram group, parents completed the PSQI and the PSQ on their children younger than age 18 years. If the Wolfram patient was 18 years or older, he/she completed the PSQI. For the control group, parents completed the PSQ for their children younger than age 18 years, and control participants 18 years or older completed the PSQI. Matching questionnaires were compared across groups.

### Disease severity variables

#### Wolfram unified rating scale (WURS) – physical

A neurologist administered the WURS [8, 19], a validated instrument developed to measure disease severity of Wolfram syndrome sequelae (e.g. vision, hearing, motor, urological, neurological, psychological and mood problems) and has been shown to have good inter-rater reliability and validity [19]. We used the physical subscale (maximum score = 160) as our measure of neurologic severity [19].

#### The physical and neurological examination for subtle signs (PANESS)

A trained examiner administered the PANESS, an age-normalized motor dysfunction assessment tool [31, 32].

#### Visual acuity

Best-corrected visual acuity was measured by Snellen optotype by a pediatric optometrist. The data were transformed into the logMAR scale for analyses [15].

### Regional brain volumes

As part of the overall natural history study, eligible patients performed MRI scans on a Siemens 3 Tesla Tim Trio at Washington University. Multiple anatomical sequences were obtained. Analyses here used data only from the T1-weighted Magnetization-Prepared Rapid Gradient-Echo (MPRAGE) sequence (sagittal acquisition, repetition time (TR) = 2400, echo time (TE) = 3.16, inversion time (TI) = 1000, voxel resolution = 1 × 1 × 1 mm, Time = 8:09 min). Regional brain volumes were extracted using Freesurfer 5.3, averaged between right and left hemispheres as appropriate and corrected for estimated total intracranial volume. The brainstem was then manually segmented into its major components: midbrain, basilar (ventral) pons, tegmentum (dorsal pons), and medulla, as previously described [9]. Brain volumes chosen for analyses were pons (ventral, dorsal, and total), medulla, and cerebellum (gray and white matter) due to their involvement in Wolfram syndrome or OSA [9, 10, 33], and association with respiration [34].

### Statistical analysis

Nonparametric statistics were used due to the small sample sizes and the non-normality of many of the sleep variables and the ranked nature of the WURS scoring. Group comparisons were performed with Mann-Whitney U tests, and correlations were performed with Spearman’s r. All statistical analyses were conducted in IBM SPSS© Version 25 (Armonk, NY).

To determine if the Wolfram group had more sleep-related problems than controls, we compared PSQ and PSQI scores across groups. To assess whether Wolfram patients with reported sleep problems were more likely to have apneas, we compared the AHI values of the subset of those with abnormal scores on the PSQ or PSQI to those with normal scores. In addition, we correlated AHI with sleep efficiency, sleep latency, sleep time, PSQI, PSQ and ESS total scores.

To assess whether Wolfram neurologic severity was related to rates of abnormal events during monitoring, we correlated AHI to WURS total score, PANESS total score and visual acuity, and to regional brain volumes known to be affected in Wolfram syndrome and involved in sleep (ventral and total pons, medulla, cerebellar gray and cerebellar white matter). Finally, we explored whether any of the other sleep study indices (CAI, MAI, OAI, and HI) correlated with these brain regions. To evaluate whether any confounding variables in Wolfram syndrome patients could explain sleep problems, we correlated AHI with age, diabetes duration, hemoglobin A1c (HbA1c; to assess recent glycemic control), and body mass index (BMI).

## Results

### Recruitment

#### Wolfram patients

Twenty-eight patients received sleep assessments (ambulatory sleep studies and/or actigraphy) in 2015 and 33 patients received sleep assessments (ambulatory sleep studies and/or actigraphy) in 2016.Twenty-seven patients obtained sleep assessments in both clinic years. Twenty-eight patients wore the ApneaLink ambulatory sleep monitoring device in 2015, 12 patients wore the Respironics Night One ambulatory sleep monitoring device in 2016, and 8 patients wore both devices. In total, 32 unique patients had one or more sleep studies. Twenty-one patients had at least 4 h of interpretable sleep study data on at least one attempt (11 from 2015 and 10 from 2016). Eleven patients’ sleep studies were not included in the analyses due to poor data quality. Four studies were scored using pediatric scoring criteria and 17 studies were scored using adult scoring criteria. Reasons for declining ambulatory sleep study testing included a prior clinical diagnosis of obstructive sleep apnea treated with continuous positive airway pressure (CPAP) nightly (*n* = 1), lack of availability of the ambulatory sleep monitoring device (*n* = 2), and anticipated discomfort with the testing (*n* = 4). Clinical sleep study records were requested for all patients and obtained and reviewed in two cases. Both patients had been diagnosed with moderate obstructive sleep apnea by in-lab sleep studies.

There were 33 individual patients who wore the Actiwatch during the 2015 and 2016 clinics, including 27 patients who wore the Actiwatch in both 2015 and 2016. Actiwatch data was selected for analyses from 2015 or 2016 to match the year that the subject had valid sleep study data, except in two cases in which Actiwatch data quality was poor during the year that sleep study data was collected.

Twenty-one patients completed both ambulatory sleep study testing and Actiwatch data collection. Of these 21 patients, mean sleep efficiency was 84% (SD = 11.6, range = 36.9–93.2), mean sleep onset latency was 35.7 min (SD = 72.6, range = 3.0–346.5), and mean sleep duration was 441.7 min (SD = 90.5, range = 220.0–624.5). 71% of the patients who completed both ambulatory sleep study testing and Actiwatch data collection were female. See Table 1 for further demographic and clinical information.

**Table 1:** Clinical, sleep, and brain volume data in Wolfram syndrome patients

	Mean	SD	Minimum	Maximum	N
Age (years)	18.0	5.8	8.9	29.7	21
Diabetes Duration (years)	12.4	6.3	0.0	26.5	21
BMI	23.2	5.0	16.1	33.0	20
HbA1c (%)	7.9	1.9	5.0	12.4	19
Apnea Hypopnea Index (AHI)	5.6	5.3	0.0	22.0	21
Sleep Efficiency (%)^a^	84.0	11.6	36.9	93.2	21
Sleep Onset Latency (minutes)^a^	35.7	72.6	3.0	346.5	21
Total sleep time (minutes)^a^	441.7	90.5	220.0	624.5	21
PSQI Total Score	5.7	3.3	1.0	12.0	18
PSQ Total Score	0.19	0.16	0	0.41	15
ESS Total Score	6.4	3.7	2.0	15.0	17
WURS Physical Score	11.1	9.7	1.0	35.0	21
PANESS Total Score	37.3	15.8	11.0	71.0	18
Visual Acuity (Logmar)	0.7	0.5	0.0	1.9	20
Ventral Pons Volume (mm^3^)	5933.9	1311.7	4316.6	7830.3	15
Dorsal Pons Volume (mm^3^)	2941.5	292.9	2563.6	3494.3	15
Total Pons Volume (mm^3^)	8875.4	1457.9	6997.6	10918.4	15
Medulla Volume (mm^3^)	2045.2	190.4	1751.0	2449.3	15
Cerebellar White Matter Volume (mm^3^)	11363.2	1651.9	8000.2	14355.3	15
Cerebellar Gray Matter Volume (mm^3^)	45064.8	4188.2	37680.2	55467.4	15

*SD* standard deviation, *BMI* body mass index, *HbA1c* Hemoglobin A1c, *PSQI* Pittsburgh Sleep Quality Index, *PSQ* Pediatric Sleep Questionnaire, *ESS* Epworth Sleepiness Scale, *WURS* Wolfram Unified Rating Scale, *PANESS* Physical and Neurological Examination for Subtle Signs. All data in Table 1 represents only Wolfram patients, and does not include controls

^a^data collected from Actiwatch

Brain MRI data was available on 15/21 of the Wolfram syndrome patients with good quality ambulatory sleep studies. This subgroup had a mean age of 18.1 years (SD = 5.9, range = 8.9–29.7 years) and was 60% female.

#### Controls

One control’s PSQ total score was an outlier (> 3 S.D. above the mean) and was therefore excluded from the primary analysis. The control group (*n* = 22) with PSQ scores was comprised of T1DM (*n* = 11) and healthy control (*n* = 11) individuals (mean age = 13.8 years [S.D. = 2.9], age range 8.6–17.8 years; 50% female). The outlier had T1DM, was 14 years old, and male. The control group (*n* = 10) with PSQI scores was comprised of T1DM (*n* = 4) and healthy control (*n* = 6) individuals (mean age = 19.6 years [S.D. = 1.6], age range 18.1–23.1 years; 70% female).

### Sleep study data (Table 2, Table 1)

The mean overall AHI was 5.6, with a range of 0–22. Five of 17 (29%) adult patients had overall AHI’s greater than or equal to 5 with a predominance of obstructive respiratory events**,** indicating obstructive sleep apnea. All 4 of 4 children aged 12 years or younger had overall AHI’s greater than or equal to 1 and obstructive AHI’s greater than or equal to 1, indicating obstructive sleep apnea in 100% of the pediatric Wolfram syndrome sample population.

**Table 2:** Descriptive statistics for sleep study indices in Wolfram syndrome patients

Index	Mean # normal / # abnormal	SD	Minimum	Maximum	N
Overall Apnea-Hypopnea Index	5.6 children: 0 / 4 adults: 12 / 5	5.3	0	22	21
Obstructive Apnea-Hypopnea Index	3.4	3.2	0.4	10.7	21
Obstructive Apnea Index	0.2	0.7	0	3	21
Central Apnea Index	2.0	3.6	0	17	21
Mixed Apnea Index	0.2	0.3	0	1	21
Hypopnea Index	3.2	3.0	0	10.6	21
Oxygen Desaturation Index	3.5	4.5	0	15.8	21

*SD* Standard deviation

One adult patient had a CAI of greater than or equal to 5 events per hour (CAI = 17/h, overall AHI = 22). A previous clinical in-lab sleep study on this patient found that this patient had moderate obstructive sleep apnea, with central apneas in the normal range. Thus, this outlier was removed from all further analyses of AHI due to this discrepancy.

### Sleep questionnaire results (Table 1, Fig. 1)

**Fig. 1:**
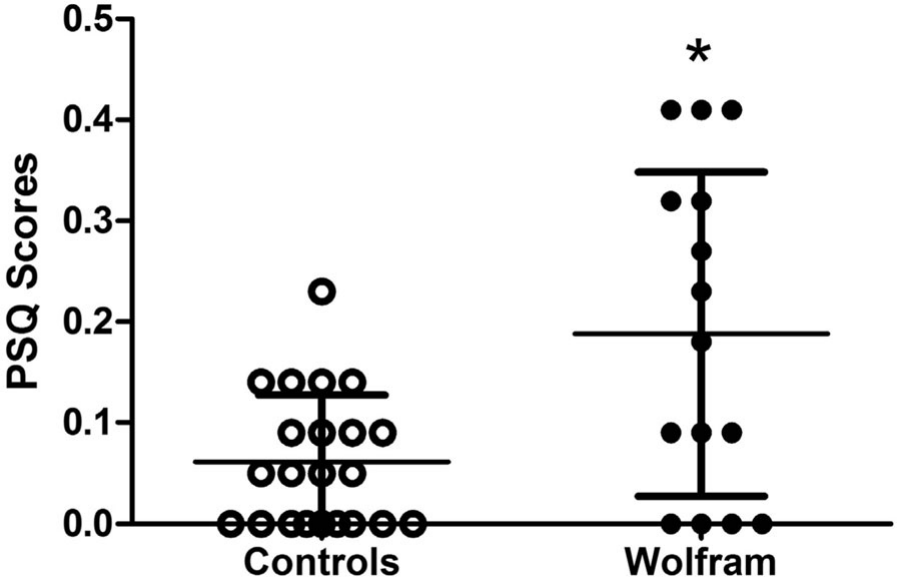
Symptoms of sleep-disordered breathing, as assessed by the Pediatric Sleep Questionnaire (PSQ). Scores are shown from Wolfram syndrome patients (solid circles) and age-matched control individuals (open circles). More symptoms of sleep-disordered breathing were reported in Wolfram syndrome patients compared to age-matched control individuals. Mean ± SD shown. *p < 0.05 compared to controls

On the PSQ, 5/15 (33%) parents of Wolfram patients and 0/22 (0%) parents of controls reported symptoms of sleep-disordered breathing (score of > 0.3). When the control outlier (PSQ score = 0.6) was included, 1/23 (0.04%) parents reported symptoms of sleep-disordered breathing. On the PSQI, 6/18 (33%) Wolfram patients and 2/10 (20%) controls reported disturbed sleep (score of > 5). Wolfram patients (mean = 0.19, [S.D. = 0.16], range = 0–0.41) had higher PSQ scores than controls (mean = 0.06 [S.D. = 0.07], range = 0–0.23; Mann-Whitney *U*, *p* = 0.02; mean ± SD for Wolfram patients and controls are shown in Fig. 1, data for Wolfram patients shown in Table 1). When the control outlier was included, Wolfram patients still had higher PSQ scores than controls (mean = 0.08 [S.D. = 0.13]; Mann-Whitney *U*, *p* = 0.05; data not shown). PSQI scores were not significantly different between Wolfram patients (Table 1) (mean = 5.7 [S.D. = 3.3], range = 1.0–12.0) and controls (mean = 4.1 [S.D. = 2.7], range = 0–10) (Mann-Whitney *U*, *p* = 0.22). Overall AHI did not differ between Wolfram patients with and without abnormal PSQ scores (for abnormal PSQ mean AHI = 5.1, S.D. = 1.6; for normal PSQ mean AHI = 4.9, S.D. = 3.6; *p* = 0.95), or PSQI scores (for abnormal PSQI mean AHI = 7.6, S.D. = 4.6; for normal PSQI mean AHI = 4.0, S.D. = 2.7; *p* = 0.10). On the ESS, 3/17 Wolfram patients had abnormal sleepiness (18%; score > 10).

### Correlations

**Fig. 2:**
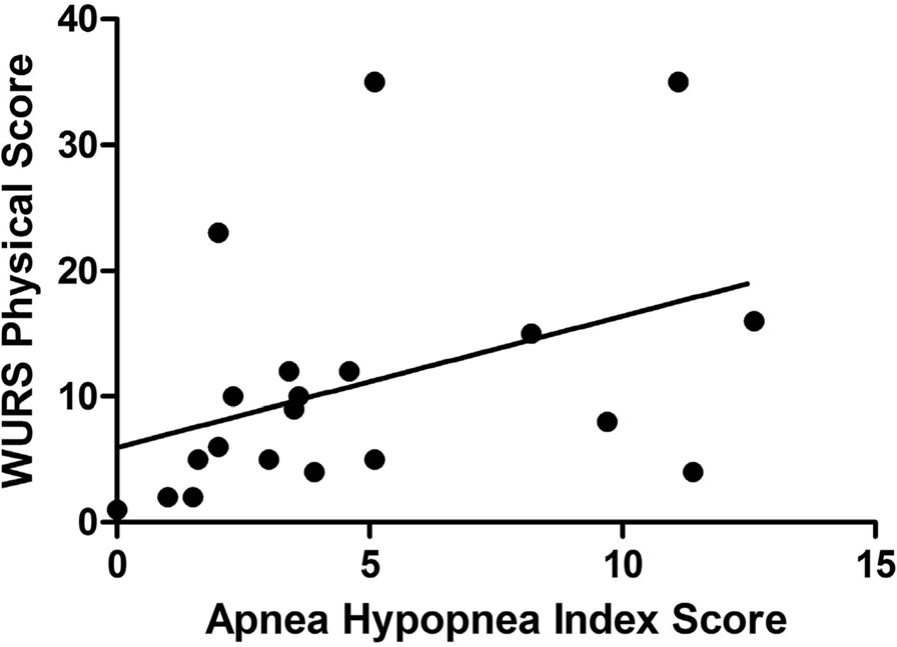
Correlation between Apnea Hypopnea Index (AHI) and Wolfram Unified Rating Scale (WURS) Physical scores. AHI was positively correlated with WURS Physical scores (*r*_*S*_ = 0.51, *p* = 0.02)

**Fig. 3:**
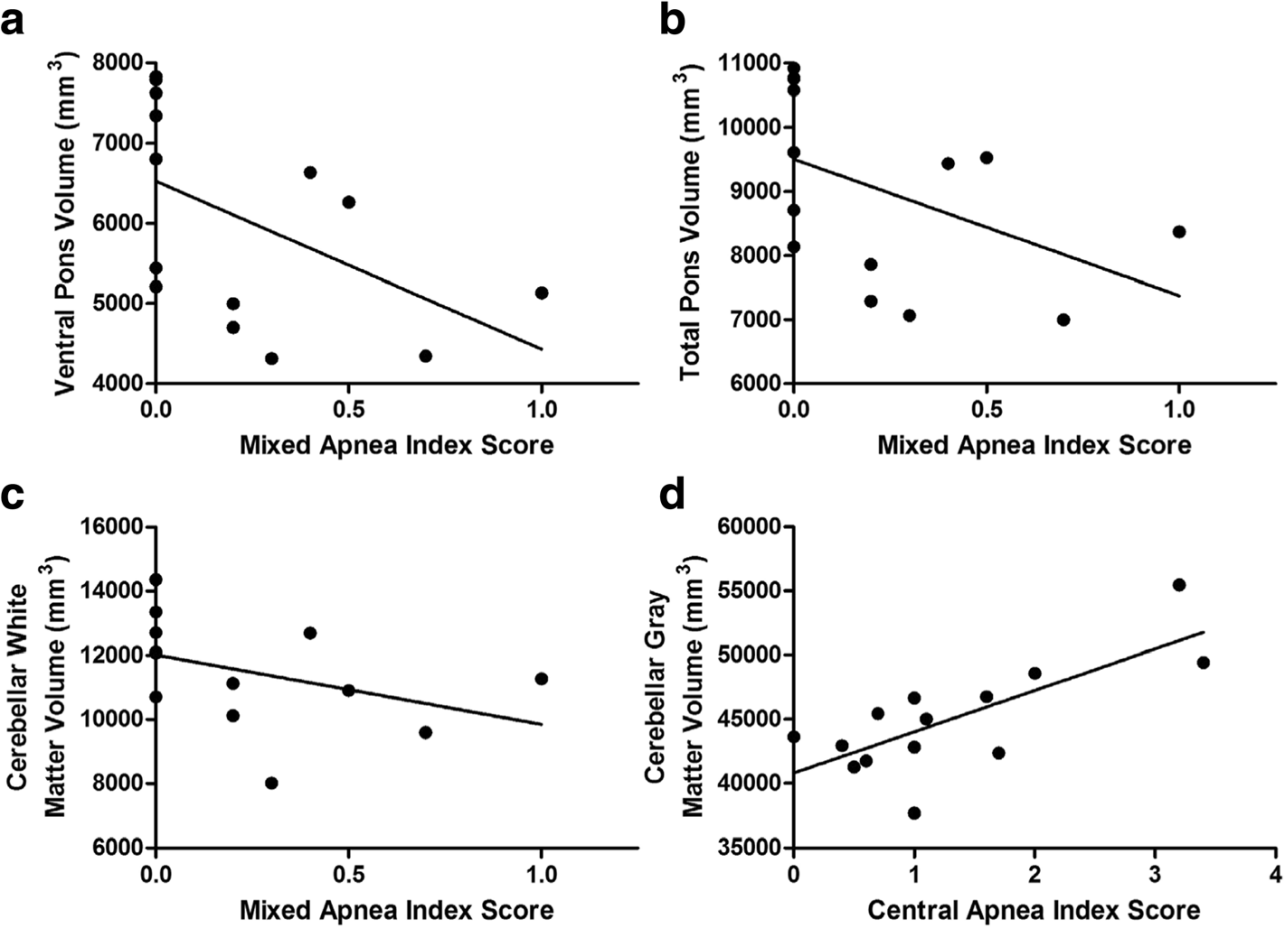
Correlations between brain volumes and sleep apnea index scores in Wolfram syndrome patients. Mixed apnea index (MAI) scores were negatively correlated with (**a**) ventral pons (*r*_*S*_ = − 0.63, *p* = 0.015), **b** total pons (*r*_*S*_ = − 0.60, *p* = 0.02, and (**c**) cerebellar white matter volumes (*r*_*S*_ = − 0.55, *p* = 0.04). **d** Central apnea index (CAI) scores were positively correlated with cerebellar gray matter volume (*r*_*S*_ = 0.62, *p* = 0.02). No *p*-value survived multiple comparisons correction

Overall AHI correlated with the WURS Physical Score such that patients with greater neurological severity tended to have higher AHI scores (*r*_*s*_ = 0.51, *p* = 0.023; Fig. 2). AHI did not correlate with visual acuity (*r*_*s*_ = 0.15, *p* = 0.54) or PANESS score (*r*_*s*_ = − 0.03, *p* = 0.90), with regional brain volumes (*p* > 0.13), sleep questionnaires (*p* > 0.29), actigraphy measures (sleep efficiency, mean sleep latency, or sleep duration; *p* > 0.36), or primary clinical measure (age, diabetes duration, HbA1c, and BMI; *p* > 0.65). There were 4 Wolfram patients that did not have T1DM. Only one had an ambulatory sleep study, which did not reveal OSA. In exploratory analyses of other sleep indices and brain regions, we found that the MAI negatively correlated with ventral pons (*r*_*s*_ = − 0.63, *p* = 0.015), total pons (*r*_*s*_ = − 0.60, *p* = 0.02), and cerebellar white matter (*r*_*s*_ = − 0.55, *p* = 0.04) volumes, such that those with more respiratory events tended to have lower volumes in these regions (Fig. 3a-c). CAI positively correlated with cerebellar gray matter volume (*r*_*s*_ = 0.62, *p* = 0.018, Fig. 3d), such that a higher central apnea index was associated with higher volume. Although these correlations are intriguing, and, for the CAI/cerebellar gray matter association, difficult to interpret, it is important to note that none of them would survive multiple comparison correction.

## Discussion

Wolfram syndrome is a complex disorder involving neurodevelopmental, metabolic, urinary, sensory, and other symptoms, many of which could interfere with sleep. This paper provides quantified objective assessment of sleep dysfunction in Wolfram syndrome, a fundamental area of investigation not reported previously. Wolfram syndrome patients in our sample had a high rate of sleep-disordered breathing, particularly OSA. Both adults and children with Wolfram syndrome had much higher rates of OSA than the general population (e.g. 29.4% vs. 2–7% [35] for adults and 100% vs. 1–5% for children [36]). An unexpected finding was that OSA was present early in the disease course. We found that all 4 of our pediatric patients met the criteria for OSA, with the youngest being only 8.9 years old. Our questionnaire results support these findings, since symptoms of OSA were endorsed by parents of children with Wolfram syndrome at higher rates than in control children. However, our pediatric sample size is too small to provide a prevalence assessment. Also, this study was underpowered for a reliable comparison of frequency of symptoms of OSA in control adults versus adult Wolfram patients. The high rate of OSA in the Wolfram syndrome population is a novel finding and may provide a clinical target for improving health and quality of life in Wolfram syndrome patients.

AHI was associated with overall disease severity as measured by a neurologist-administered clinical rating scale (WURS Physical), which is suggestive of a disease-related process underlying the presence and severity of AHI. AHI was not associated with duration of insulin dependent diabetes, glycemic control, or BMI in our sample. However, adults with type 1 diabetes (T1DM) have been shown to have a relatively high prevalence of OSA, even among non-obese adults [37]. In a meta-analysis of 22 studies, the estimated prevalence of obstructive sleep apnea (OSA) in adults with T1DM was 51.9% (95% CI = 31.2, 72.6), and mean BMI was between 22.9 and 25.8 kg/m [37], similar to our sample of WFS patients. Sleep disturbance has also been related to poor glycemic control [38]. Finally, autonomic neuropathy, as seen in diabetes, may affect control of pharyngeal muscles, contributing to increased risk of obstructive sleep apnea [39, 40]. We cannot rule out the hypothesis that insulin dependent diabetes or its complications affect sleep in Wolfram syndrome, but we did not find any relationship between diabetes duration or glycemic control and sleep disorders in our data. Thus, our present data suggest neurologic factors may be more significant. Larger samples would be needed to distinguish any additive or interactive effects between diabetes and neurologic factors in influencing sleep dysfunction in Wolfram syndrome. Due to our small sample size, we were not able to assess prevalence of OSA in Wolfram syndrome in the absence of diabetes.

Our interest in the relationships between neuropathology and sleep disturbance in Wolfram syndrome patients was driven by a number of factors. First, sleep-disordered breathing has been related to brainstem pathology in other disorders [41]. Second, sleep dysfunction can have a negative impact on the brain. Neuroimaging studies on patients with OSA have found decreased blood flow and altered white matter in cerebellum and brainstem regions [11, 12]. These regions are important in cardiovascular control and coordination of the upper airway musculature with the diaphragm [11, 42]. Third, other neurodegenerative conditions have been associated with sleep issues, which sometimes manifest among the presenting symptoms of the disease [43]. Sleep disruption itself also may contribute to disease progression in neurodegenerative conditions [43, 44]. Finally, Wolfram syndrome patients may be particularly vulnerable to the effects of sleep disruption at a cellular level, since the endoplasmic reticulum (ER) protein wolframin [1] is thought to play a role in protection against ER stress-related apoptosis [3]. Sleep disruption activates ER stress [45, 46], and in turn, ER stress in *Drosophila* has been associated with sleep fragmentation and alteration of recovery sleep, indicating a bidirectional relationship between ER stress and sleep [47]. Thus, it is possible that the neuropathophysiologic aspects of Wolfram syndrome cause or are influenced by sleep disordered breathing. Because of this background, we had hypothesized that reduced regional brain volumes in Wolfram syndrome may be related to sleep disordered breathing. While we found that higher MAI was associated with lower volumes in ventral pons, total pons, and cerebellar white matter volumes, we also found that higher CAI was correlated with greater cerebellar gray matter volume. These are intriguing findings which would need to be explored in a larger sample.

Strengths of this study include the detailed characterization of this cohort, the novel nature of this investigation, and the use of multiple instruments to assess sleep, including the interpretation of ambulatory sleep studies by a board-certified sleep specialist, actigraphy data, questionnaires, and correlations with clinical variables and brain volume data. Limitations include the small sample size. However, for a rare disorder studied with quantitative methods, the sample size is relatively large. Ambulatory sleep studies were obtained rather than in-lab sleep studies because of feasibility issues with obtaining the latter. Certain respiratory events, such as respiratory effort-related arousals and hypopneas associated with arousals from sleep, cannot be scored on ambulatory sleep studies because arousals based on electroencephalography (EEG) criteria cannot be identified [24]. Also, ambulatory sleep studies may underestimate the frequency of respiratory events since actual sleep time, as determined by EEG data, is not available on most ambulatory sleep studies [24]. Another limitation is that different brands of ambulatory sleep studies were used in the 2 clinic years of the study, due to a change in the available brand carried by the Washington University sleep center. There are missing data involving many of the parameters studied. Brain MRI data was not available on the entirety of the cohort receiving sleep assessments. However, basic demographic data did not differ significantly between those with and without brain MRI data. Ambulatory sleep study devices, actigraphy, and personnel were accessible for only a limited timeframe. Finally, data were gathered during the Wolfram research clinic during which most patients were sleeping in a hotel after traveling, which could bias our results. However, we found that sleep efficiency and sleep duration in our cohort were comparable to values found among healthy subjects, but sleep onset latency was higher in our cohort [48, 49]. In addition, self and parent reports suggest that Wolfram syndrome patients have greater sleep disturbances in their home setting compared to controls.

Future directions could include longitudinal examination of sleep issues in Wolfram syndrome and application of sleep-center based polysomnograms to provide relatively more reliable prevalence estimates of OSA in Wolfram syndrome than the ambulatory sleep study-based estimates available through this study. A larger sample size would facilitate additional exploration of the relationships among sleep issues, neuroimaging findings, and other clinical characteristics in Wolfram syndrome and could help clarify whether variations in sleep phenotypes are associated with variations in genetic mutations. The natural history of OSA in Wolfram syndrome is unclear due to the fact that our data are cross-sectional. In many neurodegenerative disorders, sleep issues are one of the heralding symptoms of disease onset [43]. Further study is required to assess whether sleep issues often arise early in the Wolfram syndrome disease course or may even sometimes present before other Wolfram syndrome symptoms manifest. Additionally, expanding the patient population beyond the age of 30 years would provide clarity on the evolution of sleep issues later in the course of Wolfram syndrome. These data in turn could help elucidate the relationship, if any, between sleep disorders and mortality in Wolfram syndrome.

## Conclusions

We have demonstrated that OSA is highly prevalent in Wolfram syndrome and that higher AHI was related to greater disease severity (higher WURS Physical scores). We have suggested that there may be neuroanatomical correlates with sleep disorders in Wolfram syndrome, since higher mixed apnea scores were related to lower brainstem and cerebellar volumes. Addressing sleep disorders in Wolfram syndrome patients and improving their sleep quality may potentially alter the natural history of the disease by mitigating ER stress and slowing cell death. An understanding of the clinical course of the sleep disorders may better clarify any co-segregation of sleep disorders with other features of Wolfram syndrome. As treatments for Wolfram syndrome are developed, their effects on sleep issues should be studied. Sleep disorders likely have considerable effects on the health and quality of life of Wolfram syndrome patients and require further attention.

## Data Availability

The datasets used and/or analyzed during the current study are available from the corresponding author on reasonable request.

## Appendix 1

We thank the former and current **Washington University Wolfram Study Group Members** for advice and support in the greater research program: P. Austin, MD (Surgery), B. Beato, BA (Psychiatry), E. Bihun, MA (Psychiatry), A. Bischoff, BA (Psychiatry), T. Doty, MA (Occupational Therapy), G. Earhart, PhD (Physical Therapy), S. Eisenstein, PhD (Psychiatry), T. Hershey, PhD (Psychiatry), J. Hoekel, DO (Ophthalmology), R. Karzon, PhD (Audiology & Comm. Sciences), J. Koller BSBME (Psychiatry), A. Licis, MD, MSCI (Neurology), H. Lugar, MS (Psychiatry), L. Manwaring, MS (Pediatrics), B. Marshall, MD (Pediatrics), A. Narayanan, BA (Psychiatry), O. Neyman, BME (Psychiatry), A.R. Paciorkowski, MD (Neurology, URMC), T. Pearson, MD (Neurology), Y. Pepino de Gruev, PhD (University of Illinois), A. Permutt, MD (Medicine) (Deceased), K. Pickett, PhD (Physical Therapy, U Wisconsin), S. Ranck, MSW (Psychiatry), A. Reiersen, MD (Psychiatry), J. Rutlin, BS (Psychiatry), J. Shimony, MD, PhD (Radiology), L. Tychsen, MD (Ophthalmology), F. Urano MD, PhD (Medicine), A. Viehoever, MD, PhD (Neurology), J. Wasson, MS (Medicine) (Deceased), N. H. White MD, CDE (Pediatrics)

## Abbreviations

AASM: American Academy of Sleep Medicine
AHI: Apnea-hypopnea index
BMI: Body mass index
CAI: Central apnea index
CSA: Central sleep apnea
DIDMOAD: Diabetes insipidus, diabetes mellitus, optic atrophy, and deafness
EEG: Electroencephalography
ER: Endoplasmic reticulum
ESS: Epworth Sleepiness Scale
HbA1c: Hemoglobin A1c
HI: Hypopnea index
ICSD: International Classification of Sleep Disorders
MAI: Mixed apneas index
MPRAGE: Magnetization-Prepared Rapid Gradient-Echo
MRI: Magnetic resonance imaging
OAI: Obstructive apnea index
ODI: Oxygen desaturation index
OSA: Obstructive sleep apnea
PANESS: Physical and Neurological Examination for Subtle Signs
PSQ: Pediatric Sleep Questionnaire
PSQI: Pittsburgh Sleep Quality Index
RERA: Respiratory effort related arousal
RST: Registered sleep technologist
SRBD: Sleep-related breathing disorder
T1DM: Type 1 diabetes
WU: Washington University
WURS: Wolfram Unified Rating Scale
